# AGING IN JAIL: CHALLENGES AND IMPROVEMENTS

**DOI:** 10.1093/geroni/igad104.3172

**Published:** 2023-12-21

**Authors:** Adriana Hernandez, Laura Driscoll, Andrea Leverentz, Jan Mutchler

**Affiliations:** University of Massachusetts Boston, Boston, Massachusetts, United States; Boston University, Boston, Massachusetts, United States; North Carolina State University, Raleigh, North Carolina, United States; University of Massachusetts Boston, Boston, Massachusetts, United States

## Abstract

The increase in incarcerated older people has created challenges in correctional facilities, including settings ranging from large prisons to smaller county jails. Aging while incarcerated brings a new dimension of difficulties for older people, and to the correctional system as a whole. The resources and environments are unsuitable for an aging population and ultimately result in greater health complications. The COVID-19 pandemic exacerbated existing challenges experienced by older adults. To further assess the issues that incarcerated older people face, researchers from UMass Boston partnered with a local jail to determine the current needs and deficiencies experienced by older men. Focus groups were conducted in person with incarcerated older men regarding their experiences, opinions on having a unit specifically for older adults, and institutional changes that occurred during the COVID-19 pandemic. Zoom interviews were also carried out with staff. These conversations suggest that older incarcerated men encounter unique conditions that challenge their well-being. Such challenges include limited access to medical care and proper diets, lack of environmental accommodations, and inadequate exercise opportunities. These challenges exacerbate the burden of being in jail and can lead to higher risks of chronic illness. As well they have potential implications for the communities to which the men are released. Older men and jail staff were receptive to creating a unit specifically for older adults as a means of remedying some of these challenges. This poster offers policy suggestions to improve correctional facilities that better align with the unique needs of incarcerated older people.

